# The History and Capabilities of Herbal Simples

**Published:** 1890-05-03

**Authors:** W. T. Fernie


### The History and Capabilities of Herbal Simples.

By W. T. Fernie, M.D.

*(Continued from page 6.)*

### VIII.—BLACKBERRIES—BROOKLIME.

### Blackberries.

Are the well known fruit of the common bramble, which grows in every English hedgerow. The name of the bush is derived from brambel, or brymbyll, signifying "prickly." Botanically it is termed Rubus fracticosus, and it belongs to the rose order of plants. Blackberries are often called bumble kites, from bumble, the voice of the bittern, as in Chaucer; and kite or kyte, a Scotch word for belly; the name bumble kite being applied, says Dr. Prior, or from the rumbling and bumbling caused in the bellies of children who eat its fruit too greedily." The ancient Greeks gave brambleberries for gout. In England the bramble has long been esteemed as a powerful astringent. Its leaves are gathered in the Spring and dried, then when required, a handful of these leaves is infused in a quart of boiling water, and the infusion when cool is drank freely. Chemically the bark of the bramble-bush is found to contain a considerable quantity of tannin. The blackberry fruit contains malic and citric acids, pecten, and albumen. It has also acquired the name of scaldberry, from producing, as some say, scaldhead in children who devour the fruit to excess, or as others declare, from the supposed curative effects of the leaves, and berries in that disease of the skin; or, again, it may be from the use of the leaves as applied to scalds.

Cornish people say that the first blackberry of the year will banish warts. A decoction of the root is used to cure whooping cough in its second stage; this is also useful for summer, or infantile diarrhœa. An ounce of the bruised root should be boiled in three half pints of water down to a pint, and a small teacupful may be given every three or four hours. Gerard says about bramble leaves they "heal the eyes that hang out, and stay the hemorrhoides, if they can be laid there unto." The London Pharmacopœia, 1696, declared the ripe berries of the bramble to be a great cordial, and to contain a notable restorative spirit. As soon as over-ripe they immediately become quite indigestible. In Cruso's treasury of easy medicines, 1771, it is directed for old, inveterate ulcers, "take a decoction of blackberry leaves, made in wine and foment the ulcers with this, hot, each night and morning, which will heal them however deep and difficult to be cured." In Somersetshire and Sussex, country people are unwilling to gather blackberries after Michaelmas day, as then no longer good. They say the devil goes round on October 11., Michaelmas old style, to spite the saint, and spits on the blackberries, so that if anyone eats them after that time, he or she would die, or have great trouble before the year was out. A very agreeable wine may be made from the blackberry fruit; and blackberry jam is used in many rustic homes as a remedy for sore throats. In olden times the fruit and the flowers of the bramble were considered efficacious against the bite of a serpent. In Devonshire the peasantry still think that if anyone is troubled with pimples, or blackheads—small boils—he may be cured by creeping on the hands and knees from east to west, nine times beneath an arched bramble-bush, which has grown into the earth at both ends. This is evidently a relic of the old Dryad superstition, when the angry deities of certain diseases, who inhabited particular trees, had to be appeased before their special diseases could be cured. We may profitably remember the parable told in the Old Testament, Judges, chapter ix., verses 8-15, of the trees choosing the bramble for a king, when the olive, the fig-tree and the vine had excused themselves from accepting that dignity. With regard to the prevailing popular notion that blackberries may be allowed when other fruits and vegetables are for the time forbidden, because of summer diarrhœa, this is based on the mistake of attributing to the fruit the well-known astringency which is rather possessed by the roots and bark of the bramble. Tom Hood, in his humorous way, facetiously described a negro funeral as "going a black burying."

### Brooklime.

Of all celestial colours which adorn our wild flowers, by far the most "darkly, deeply, beautifully blue" is that of the Speedwells, which include within their ranks the common Brooklime, or Veronica beccabunga, of our English brooklets and small streams. This plant belongs to the natural order of Scrophularineæ, or Scurvy-curers; and it is an established simple against scorbutic or cutaneous diseases.

The name Beccabunga is derived from beck, in provincial English, a brook; and pungen, to smart, because of its pungency. The prior name, Veronica, has a much more poetical and exalted significance. The legendary tale, says Mr. Grant Allen, attached to these humble friendly Veronicas is wonderfully pretty and touching. We all remember how the napkin with which a compassionate maiden wiped the face of Christ on the morning of His crucifixion bore for ever after the imprint of the divine features. This miraculous portrait thus preserved as the one and only true picture of our Lord, was known by a term of mongrel Greek and Latin, as the "vera eikon," the veritable likeness, or image of the martyred Saviour. By an easy transposition of sound and sense, the unknown maiden was popularly canonised as St. Veronica. Then, at some later period, by a graceful conceit, the botanist Tournforte transferred the title of Veronica to the exquisitely blue blossoms of the speedwells, because they seemed to exactly mirror in their delicate hue the brilliant azure colour of the sky above. Each speedwell was the *vera icon* of the open heaven, and so it was happily named Veronica. But, alas, for the poetry of this pious thought. By the stern prose of facts we are assured far more reliably that the petals of the speedwells are so transcendently blue only to attract the eyes of fertilising insects, and are streaked with dainty darker lines to guide these useful visitors straight to the further allurement of honey which they are seeking. The lovely but fragile petals fall off at a touch; whence comes the name Speedwell, or Farewell, or Good-bye, or Forget-me-not, because the fleeting corolla flies away as soon as the flower is gathered. The leaves and stems of the brooklime are slightly acid and astringent. Chemically, they contain a bitter principle, and some tannin. Formerly the plant was ordered by the London Pharmacopœia as an ingredient of the compound succus of scurvy-grass. The juice of brooklime in a full dose is an easy purge. "Copia sumptus, alvum movet commodissime." The leaves are often sold with water-cress, and may be readily mistaken for that plant; but the true watercress has neither the hollow stalks, or the serrated leaflets of brooklime. Gerard says, "The leaves of brooklime boiled, and stamped with hog's grease into the form of a cataplasm, mightily defendeth wounds, so that no humor or accident shall happen thereunto. The leaves of brooklime and the tendrils of asparagus eaten with oyle and vinegre do helpe the strangurie and stone. Being fryed with butter and vinegar, and applyed warme, it helpeth all manner of tumours and swellings." If taken with, or in place of water-cresses, the leaves and the juice of brooklime should be eaten freely, as the powers of the plant are inconsiderable. But it better suits certain stomachs—"What cures Sancho makes Martha sick."

